# The Influence of Police Related Media, Victimization, and Satisfaction on African American College Students' Perceptions of Police

**DOI:** 10.3389/fsoc.2019.00065

**Published:** 2019-09-10

**Authors:** Andrew Sheldon Franklin, Robert Kelvin Perkins, Morgan D. Kirby, Kijana P. Richmond

**Affiliations:** ^1^Department of Psychology, Norfolk State University, Norfolk, VA, United States; ^2^Department of Sociology, Norfolk State University, Norfolk, VA, United States; ^3^Department of Mass Communications and Journalism, Norfolk State University, Norfolk, VA, United States; ^4^Department of Psychology, Florida State University, Tallahassee, FL, United States

**Keywords:** victimization, perception of police, college students, influence of media and law enforcement, African American

## Abstract

This study explored the roles of police related television programming, and satisfaction with most recent police contact in predicting perceptions of law enforcement performance and treatment of minorities for students with no police victimization experiences. The current study also explored the role of personal and familial police victimization experiences impact on perceptions of police. A convenience sample of 246 African American students (168 females and 78 males) ranging in age from 17 to 59 (M_age_ = 21.73), attending a historically Black university completed a questionnaire assessing demographic information, satisfaction with most recent contact with police, personal, and familial police victimization experiences, and law enforcement related television programming. Results showed that African American students with personal and familial police victimization experiences had significantly different perceptions of law enforcement than individuals with no victimization experiences or just familial victimization experiences. Results also highlighted the predictive power of crime reality shows, police excessive force media consumption, and satisfaction with police in influencing perceptions of police. These significant relationships and causal models may be salient for understanding pertinent factors that influence perceptions of law enforcement in African American college students.

## Introduction

The mission of law enforcement involves police performing five distinct responsibilities: preserving the peace, protecting people and property, investigating crimes and arresting offenders, preventing crime from occurring, and enforcing laws (Bouza, 1990). By the 1970s, community policing was a focus of police departments and involved law enforcement collaborating with communities to assist them with unique problems in an effort to fulfill the mission (Stevenson, 2008). Unfortunately, law enforcement–minority relations continue to be problematic in the twenty-first century, which makes it difficult for police to fulfill their mission and hinders the quality of life of all parties involved. One of the reasons the relationship between law enforcement and minority groups may be strained is because minority groups (i.e., behavioral minority groups and racial minority groups) tend to report psychological and emotional abuse as a routine part of their interactions with police (Cox, 1996). Though unhealthy dynamics may occur between law enforcement and minorities in general for a variety of reasons, there is a substantial history of negative attitudes toward law enforcement held by African Americans when compared to other racial and ethnic groups (Apple and O'Brien, 1983; Priest and Carter, 1999; Brown and Benedict, 2002). For example, Pastore and McGuire (2001) found that White Americans perceive policing as fair and balance, while some racial minorities view policing as oppressive and life-threatening. Even more thought-provoking is that research also shows that White college students are less likely to feel victimized by law enforcement than African American college students (Walker et al., 2007; Ranapurwala et al., 2016; Girgenti-Malone et al., 2017).

Police perceptions are of extreme importance when studying the relationship between minority and law enforcement, because ultimately police cannot be effective without public support. A lack of support from the public would render the police unable to apprehend criminals, deficient of information to solve crimes, uninformed about crimes, and unable to recruit quality recruits with diverse backgrounds (Cox, 1996). African American students' perceptions of police should be explored in a collegiate environment given the projected demographic changes occurring in the coming decades (i.e., a dominant multi-cultural majority) and the scant research literature focusing on the perceptions of law enforcement held by African American college students. The authors of this study were only able to find a couple of research articles exploring the perceptions of law enforcement held by African American college students (Chow, 2010; Davis, 2014).

There are many things that influence African Americans' public perception of police, including victimization experiences. Researchers have found that crime victimization and police victimization have the same impact on the victims (Hanson et al., 2010). Police victimization is the act of police officers using their systematic power to unlawfully harass, oppress, and/or abuse citizens that leads to undue stress. In the 80s and 90s, police harassment simply meant that African Americans were more likely to be stopped and questioned by the police (Cobbina et al., 2016); however, more modern experiences have shown that African Americans have suffered beatings and even murder at the hands of police (Ranapurwala et al., 2016). There is very little literature that focuses on the impact of improper police conduct on African American college students' perceptions of police. In general, positive perceptions of police officers tend to be experienced by African Americans when their interactions with law enforcement are respectful and non-threatening (Birzer, 2008). Negative perceptions of law enforcement may abound when an individual has contact with law enforcement that is characterized by rudeness, apathy on behalf of the officer, and a lack of desire to take care of the problem (Birzer, 2008). Given the aforementioned research literature, perceived, and actual victimization experiences may differ based on the party that is completing the assessment (e.g., public and police officer) and the part of the interaction that is the focal point (e.g., what officers do vs. how they do it). When individuals are void of victimization experiences, they may be influenced by familial victimization experiences. Negative experiences that family members had with law enforcement may influence an individual's perception of law enforcement in ways similar to that of personal experiences.

When examining experiences with law enforcement one must take into consideration the notion of procedural justice and differing perceptions between law enforcement and the public at times. Procedural justice focuses on how law enforcement interacts with the public; it is a necessary precursor to perceived legitimacy, which influences police efficacy and public safety (Peterson et al., 2017). Procedural justice consists of four components: voice, transparency, impartiality, and fairness. The voice component deals with residents being able to voice their perspective and be heard in the interaction (Tyler, 2004). Transparency involves law enforcement authorities sharing processes, policy, and procedure throughout the interaction while maintaining confidentiality when necessary. Impartiality involves police making decisions based on legal facts and an objective evaluation of the situation. Fairness is about community members being treated with dignity regardless of their situation (Tyler, 2004). Meares et al. (2015) concluded that there is a disconnect between how the public views their actions and how law enforcement assesses their own actions during police contacts. Some of this may be due to aggressive police tactics, which Black and White individuals alike have reported in Gau and Brunson's (2010) research. It should be noted that the rejection of police behavior by the public may be based on fairness, as opposed to the law, given that the public's familiarity with the law is sometimes low (Meares et al., 2015). Ultimately, the notion of procedural justice affects individuals' confidence in police as shown in recent statistics highlighting 42% of White Americans having high confidence in their local police department in contrast to only 14% of Black Americans exhibiting that same level of confidence (Morin and Stepler, 2016). In an effort to not stereotype or overly generalize, there are interactions with Black Americans and law enforcement that are characterized by a great deal of respect, satisfaction, and presence of procedural justice. Nevertheless, procedural justice must be considered in face to face interactions and multimedia that depicts law enforcement given that it may influence attitudes toward law enforcement.

Multimedia, specifically television programming, deserves much exploration in the assessment of African Americans' perception of law enforcement because media influences perceptions of law enforcement when individuals lack personal experiences (Cox, 1996; Maxson et al., 2003). Law enforcement officers are often portrayed in news coverage programs, crime-based reality television shows, crime dramas, and newspapers. The way that law enforcement is depicted across these media tends to vary. Law enforcement is often depicted in news programs positively due to an overview of a crime and a focus on an arrest that usually portrays the law enforcement officer as the heroine (Sacco and Fair, 1988; Iyengar, 1991). Crime-based reality television shows are factual, tend to focus on actual crime, and overemphasize the arrests made by law enforcement officers; given that law enforcement usually has complete control over how they are depicted, they are often seen as heroic in crime-based reality television shows, even when using excessive force as a means to pursue justice (Oliver, 1994). Television crime dramas are fictional, but may be based on true stories at times, and depict law enforcement officers in a positive light. In some of these shows, law enforcement officers act as vigilantes and violate civil liberties and laws to pursue justice (Sparks, 1995). Additionally, most crimes that are presented in crime dramas are solved, which is not true to real life but necessary for a plot line that needs a resolution for entertainment purposes. Individuals watching a show to be entertained will be less likely to be influenced by what they see than an individual who is watching a broadcast for information (Valkenburg and Patiwael, 1998). Additionally, perceived realism and the purpose behind watching a broadcast seems to be a key component of media influence (Potter, 1986; Valkenburg and Patiwael, 1998). Some research has posited that college students' perceptions are less affected by mass media depicting law enforcement due to a focus on equity and fairness and acquired criminal justice knowledge (Engel, 2005; Mbuba, 2010).

If the information about law enforcement communicated via television indicates that procedural justice has been neglected, then this is likely to have a negative impact on the public's perception of law enforcement (Sunshine and Tyler, 2003). However, if the media depicts law enforcement positively, such as is often the case with television news and crime-based reality television, increased confidence in law enforcement is likely to result when individuals do not have an arrest record or were not victimized (Callanan and Rosenberger, 2011). Weitzer (2002) found that negative perceptions of law enforcement are likely to increase and be pronounced when accounts of law enforcement misconduct are highly publicized. Historically, an example of this can be seen in the drop of law enforcement approval ratings of Los Angeles citizens following the widespread coverage of the 1991 videotaped beating of Rodney King and the 1979 fatal shooting of Eulia Love (Weitzer, 2002). Additionally, the coverage of police misconduct in the deaths of Michael Brown in August 2014, Tamir Rice in November 2014, Alton Sterling in July 2016, Freddie Gray in April 2015, and many others have far reaching consequences on American citizens' mental health, emotional health, physical health, spiritual health, and perceptions of law enforcement.

## Theoretical Justification for Sample

American social institutions may be perceived by racial groups in different ways, and Blacks and Whites serve as an example of contrasting perceptions of law enforcement. Race is one of the most important predictors of attitudes toward police and criminal justice institutions. Our selection of an African American student population is informed by the group-position model of race relations, which is a variation of conflict theory. Group-position theory views racial animus as a reflection of group competition and conflict over material items, power, and status in a multi-racial society (Blumer, 1958). In this theory, dominant group interests are predicated on radical beliefs that they have claims to scarce resources and a desire to defend their interests against minority groups. Minority groups in this model are interested in obtaining a greater share of goods and are motivated by unfair and exclusionary treatment by the dominant group. Weitzer and Tuch (2005) extended the group-position thesis to the analysis of group relations in social institutions. If a dominant group believes that it is entitled to valuable resources it is safe to assume that the dominant group will have a natural affinity for social institutions, like law enforcement, that serve their interests. African Americans should be more inclined to view the police as contributing to their subordination through legal and illegal practices, which may involve mistreatment of minorities and a lack of regulation in policing. This study focuses on an African American student population given the group-position thesis, yet it does not offer direct comparisons of racial groups or assume that African Americans are anti-police.

## Purpose

The dynamic between law enforcement and African Americans has been perceived by some African American students as characterized by a lack of respect, a lack of procedural justice, absent distributive justice, a refusal of compliance, anxiety from both parties, and a host of other factors (Brown and Benedict, 2002; Engel, 2005; Mbuba, 2010; Girgenti-Malone et al., 2017). As previously highlighted, police victimization, and television programming may prove to have a significant influence on African American college students' perceptions of law enforcement. In the absence of experiencing firsthand victimization on behalf of law enforcement, incidents involving inappropriate and excessive force used by police officers become legend in minority communities and further the negative image of police already present (Cox, 1996; Chermak et al., 2006). The general public is informed about incidents involving law enforcement and African American relations via media, family members, friends, and through their own experiences.

The importance of the public's perception of law enforcement cannot be understated due to its enmeshment with perceived legitimacy of law enforcement. If law enforcement is not viewed as legitimate then African Americans will be less likely to cooperate with law enforcement and support policies that empower law enforcement (Sunshine and Tyler, 2003). Additionally, increased criminality could ensue due to an unwillingness to call law enforcement when in need, and behavior that challenges structures and agents of authority could continue. There is a lack of literature examining college student perceptions of law enforcement and the factors that are related to the phenomenon (Chow, 2010; Davis, 2014). An exploration of the significance of police victimization in various forms and television programming consumption can serve as a means to facilitate dialogue between college students and campus police and develop intervention and policy to increase the likelihood of a healthy dynamic between law enforcement and the campus community. The researchers have the following hypotheses:

H1: There will be a significant difference in perceptions of police performance across the law enforcement victimization groups (i.e., family victimization, personal victimization, and personal and familial victimization) and the control group (i.e., no victimization experiences).

H2: There will be a significant difference in perceptions of police treatment of minorities across the law enforcement victimization groups (i.e., family victimization, personal victimization, and personal and familial victimization) and the control group (i.e., no victimization experiences).

H3: Fictional crime drama media, crime reality (i.e., non-fictional) show media, police use of force media (e.g., fictional or non-fictional) will be significantly related to perceptions of law enforcement performance and treatment of minorities for African American college students with no police victimization experiences.

H4: Satisfaction with police will be significantly related to perceptions of law enforcement and treatment of minorities for African American college students with no police victimization experiences.

## Methods

### Participants

This study was approved by a southeastern United States university's Human Subjects Institutional Review Board. All the human subject protections were followed throughout the duration of this research project. Also, informed consent was obtained from all the participants in writing and orally.

A convenience sample of students who voluntarily consented (*N* = 287) from a southeastern university with a historically Black college and university (HBCU) distinction were recruited for this study. Enrollment at the institution and active class registration served as eligibility criteria to participate in the study. Some of the participants were compensated with extra course credit toward an in-class assignment offered by their instructor. Participants were also given the opportunity to participate in a lottery for a gift card worth $25 for their participation. This study is solely focused on predictors of law enforcement perceptions for college students who identify as African American; all non-African Americans were excluded from the analyses (*n* = 41), thus making the sample size 246. Additionally, for the regression analyses, the sample was condensed to 180 to focus solely on African American college students that did not have police victimization experiences.

Most of the sample identified as female (168), and the mean age for this sample was 21.73, ranging from 17 to 59. The majority of the sample was academically classified as sophomores (29%). The majority of the sample indicated an income of “$20,000–30,000” (10%), and most of the individuals in this sample politically identified as a Democrat (76%).

### Measures

#### Demographic Questionnaire

Personal information was collected using a series of closed-ended questions, including gender, age, academic classification, income, and political affiliation. These demographic variables served as a means of describing the sample and were not utilized in major analyses.

#### Perceptions of the Law Enforcement

The perceptions of law enforcement scale is an eight-item measure that comprises two scales. The measure assesses confidence in police performance and treatment of minorities. Participants were asked the extent to which they agreed or disagreed with such items as “The law enforcement will only use lawful means to combat crime” and “The law enforcement are more likely to use physical force against minority people than Whites.” As reported in Chow's (2010) initial validation study, the two dimensions of perceptions of law enforcement in this study exhibited Cronbach alphas ranging from 0.791 to 0.854.

#### Contact With Law Enforcement

An item assessed participants' satisfaction with performance based on their most recent official contact with the law enforcement in either emergency or non-emergency situations. Participants utilized a 5-point scale (1 = very dissatisfied to 5 = very satisfied) for their response.

#### Law Enforcement Victimization

An item assessed familial or personal experiences of harassment or mistreatment by the law enforcement in the past 12 months prior to the survey. Participants utilized a 4-point scale (0 = No Victimization to 3 = Personal and Family Victimization) for their response (Chow, 2010).

#### Media Variables

The analyses included three types of media consumption: crime dramas, crime-reality shows, and media that involved an unarmed man suffering excessive physical force on behalf of a law enforcement officer (i.e., non-fictional). The crime dramas scale captures frequency of viewing Law and Order, Homicide, and NYPD Blue. The crime-reality show scale measures frequency of viewing Cops, American Justice, America's Most Wanted, and Justice Files. All the questions gauging viewership of these programs were originally coded on a five-point Likert scale (1 = Never to 5 = Almost Every Day).

### Procedures

The participants completed instruments in paper-pencil format and online through a secure web database as part of a university institution review board-approved study. The study was advertised as an investigation of personal perceptions of law enforcement, and students taking courses in the Departments of Psychology, Sociology, and Mass Communications and Journalism were offered the opportunity to participate. The students who chose to participate in the study received and then read a brief introduction about the project including the nature of the study, topics of some questions to be answered, and a statement informing the reader that all participation was voluntary with an option of withdrawing at any time.

## Results

A statistical power analysis was performed with GPower for sample size estimation (Faul et al., 2007). The effect size in this study was 0.15, considered to be a medium using Cohen's (1988) criteria. With an alpha = 0.05 and power = 90, the projected sample size needed with this effect size is ~*N* = 147. Thus, our sample size of 246 participants will be more than adequate for the main objective of this study involving predicting confidence in law enforcement and treatment of minorities.

A statistical power analysis was also performed with GPower for sample size estimation for our model predicting emotional well-being from law enforcement perceptions, victimization, satisfaction, and forms of media consumption (Faul et al., 2007). The effect size for this analysis was 0.15, considered to be medium using Cohen's (1988) criteria. With an alpha = 0.05 and power = 90, the projected sample size needed with this effect size is ~*N* = 157. Thus, our sample size of 246 participants will be more than adequate for predicting the emotional well-being from confidence in law enforcement, perceptions of treatment of minorities, satisfaction with law enforcement, victimization, and two control variables.

Principal axis factor analysis was conducted with varimax rotation to assess the underlying structure for the 10 items of the *Perceptions of Law Enforcement* questionnaire. The assumption of independent sampling was met and the assumptions of normality, linear relationships between pairs of variables, and the variables' being correlated at a moderate level were checked. Three components were rotated, based on the eigenvalues over one criterion and the scree plot. After rotation, the first component accounted for 31% of the variance and the second component accounted for 20% of the variance. These findings seem similar to Chow's (2010) findings after computation of a factor analysis on *Perceptions of Law Enforcement*. Table 2 displays the items and component loadings for the rotated components, with loadings <0.32 omitted to improve clarity. The first factor seems to index “confidence in law enforcement performance” and consists of five items. The second factor seems to index “law enforcement treatment of minorities” and consists of two items as the third item was eliminated because it did not address treatment of minorities. The elimination of the third item was consistent with Chow's (2010) approach with the instrument. “Law enforcement treatment of minorities” was named as a factor because the items were highly correlated with each other. As a general guide, factors that have <2 items should be interpreted with caution and can be considered reliable if they are highly correlated with each other and uncorrelated with other variables (Tabachnick and Fidell, 2007). Cronbach alpha reliability coefficients for the law enforcement performance and treatment of minorities scale were 0.791 and 0.854, respectively.

Descriptive statistics for the media, victimization experiences, and law enforcement perception subscales are provided in Table 1. Mean scores for satisfaction with most recent contact with law enforcement were neutral. Law enforcement victimization experience seems to indicate that most of the sample has a family member who endured law enforcement victimization. On average the sample views fictional crime drama media a few times a month. This sample viewed crime reality media that depicted law enforcement between a few times a month to a few times a year. Media that involved usage of excessive physical force by police officers on an unarmed individual was viewed by this sample a few times a month to a few times a week. Overall, this sample of university students was neutral to somewhat in disagreement as far as their confidence in police to do their jobs. Treatment of minority scale indicated that on average the sample somewhat agreed that law enforcement was more likely to use physical force against minorities and aboriginal individuals than Whites.

**Table 1:** Descriptive statistics for satisfaction with law enforcement, victimization, law enforcement media consumption, emotional well-being, and law enforcement perception variables.

	***N***	**Minimum**	**Maximum**	**Mean**	***SD***	**Median**	**Mode**
Contact with law enforcement	230	1	5	3.04	1.12	3	3
Law enforcement victimization	246	0	3	1.13	1.01	1	1
Fictional crime drama media	246	1	5	3.29	1.29	3	4
Crime reality media	245	1	5	2.52	1.22	2	2
Excessive force media	243	1	5	3.43	1.16	3	3
Perception of police performance scale	242	1	5	2.50	0.76	2.40	2
Treatment of minority scale	242	1	5	4.06	1.16	4.50	5

*Contact with law enforcement utilized a 5-point scale (1, very dissatisfied to 5, very satisfied). Law enforcement victimization utilized a 4-point scale (0, No Victimization, 1, Familial Victimization, 2, Personal Victimization, and 3, Personal and Family Victimization). Fictional Crime Program Consumption, Crime Reality Program Consumption, and Police Excessive Force Media Consumption utilized a 5-point scale (1, Never, 2, A Few Times A Year, 3, A Few Times A Month, 4, A Few Times A Week, and 5, Almost Everyday). Perception of Police Performance and Treatment of Minority utilized a 5-point scale (1, Strongly Disagree, 2, Somewhat Disagree, 3, Neutral, 4, Somewhat Agree, and 5, Strongly Agree)*.

**Table 2:** Rotated factors for police perception scale.

	**Factors**		
**Item**	**1**	**2**	**Communality**
Officers are usually fair.	0.859		0.194
The law enforcement do a good job of stopping crime.	0.696		0.406
Officers are usually courteous.	0.686		0.341
Law enforcement always respond promptly when called.	0.535		0.620
The law enforcement will only use lawful means to combat crime.	0.448		0.596
The law enforcement are more likely to use physical force against minority people than Whites.		0.915	0.260
The law enforcement are more likely to use physical force against aboriginal (native) people than Whites.		0.820	0.579
The law enforcement spend most of their time going after people who commit petty crimes and ignore most of the bad things going on.		0.592	0.497
Eigenvalues	2.92	1.99	
% of Variance	30.78	20.38	

*Loadings <0.32 are omitted*.

The researchers computed a one-way ANOVA comparing the perceptions of police performance scale of participants who had four different police victimization experience backgrounds. A significant difference was found among the police victimization groups [*F*_(3, 238)_ = 3.696, *p* < 0.05]. Tukey's HSD was used to determine the nature of the differences between the police victimization groups. This analysis revealed that students with personal and family police victimization experiences (*m* = 2.17, sd = 0.64) had lower scores than students who had no police victimization experiences (familial or personal) (*m* = 2.62, sd = 0.82) and students with just family police victimization experiences (*m* = 2.56, sd = 0.75).

The researchers computed a one-way ANOVA comparing the perceptions of police treatment of minorities scale for participants who had four different police victimization experiences. No significant differences were found [*F*_(3, 238)_ = 2.465, *p* > 0.05]. The students did not differ significantly in their perceptions of how police treat minorities across the different police victimization backgrounds.

A multiple linear regression was calculated to predict non-victimization students' perceptions of police performance based on satisfaction with police in most recent contact, crime reality show consumption, crime drama show consumption, and police excessive force media consumption (See Table 3). The overall regression, including three of the four predictors, was statistically significant, *R* = 0.37, *R*^2^ = 0.13, adjusted *R*^2^ = 0.11, *F*_(4, 157)_ = 6.092, *p* < 0.01. Approximately 13% of the variance in perceptions of police performance could be accounted for by satisfaction with most recent contact with police, crime reality show consumption, and police excessive force media consumption. The significant predictors were positively related to perceptions of police except for police excessive force media consumption, which was negatively related to the dependent variable. Crime reality show consumption is the strongest unique predictive contribution in this model.

**Table 3:** Variables predicting confidence in law-enforcement performance.

	**Unstandardized coefficients**					***r***	
**Variable**	***B***	**Standard error**	***B***	***T***	***p***	**Semi-partial**	**Tolerance**
Satisfaction with police	0.139	0.055	0.196	2.548	0.012	0.189	0.937
Fictional crime drama media	0.021	0.053	0.035	0.396	0.693	0.029	0.724
Crime reality media	0.148	0.056	0.242	2.672	0.008	0.198	0.672
Excessive force media	−0.119	0.056	−0.169	−2.139	0.034	−0.159	0.888

A multiple linear regression was calculated to predict non-victimization students' perceptions of police treatment of minorities based on satisfaction with police in most recent contact, crime reality show consumption, crime drama show consumption, and police excessive force media consumption (See Table 4). The overall regression equation was not significant *R* = 0.20, *R*^2^ = 0.04, adjusted *R*^2^ = 0.02, *F*_(4, 157)_ = 1.68, *p* > 0.05. None of the predictors can be used to predict non-victimization students' perceptions of police treatment of minorities in this model.

**Table 4:** Variables predicting treatment of minorities.

	**Unstandardized coefficients**					***r***	
**Variable**	***B***	**Standard error**	***B***	***T***	***p***	**Semi-partial**	**Tolerance**
Satisfaction with Police	−0.102	0.084	−0.098	−1.215	0.226	−0.095	0.937
Fictional Crime Drama Media	−0.022	0.081	−0.025	−0.272	0.786	−0.021	0.724
Crime Reality Media	−0.047	0.085	−0.052	−0.547	0.585	−0.043	0.672
Excessive Force Media	0.165	0.085	0.160	1.934	0.055	0.151	0.888

## Discussion

Understanding the factors related to African American college students' perception of law enforcement is extremely pertinent given the role that those perceptions could play in police evaluations, policy formation and implementation, and the need for collaborative efforts between officers and the public to ensure safety and optimal life in communities. The current study investigated significant differences in police perceptions that may result from personal and familial police victimization or lack thereof. Additionally, the current study examined the predictive role of four variables on confidence in law enforcement performance and perceptions of how law enforcement treats citizens of minority groups.

Support for significant differences in perceptions of police performance across levels of police victimization was found in this study. African American college students who had personal and familial experiences of being harassed or mistreated by police had significantly lower confidence in police than students who had no police victimization experience and individuals who had family members who endured law enforcement victimization. Given that personal and familial police victimization experiences were significantly different from both non-police victimization groups, but not significantly different from just the personalized police victimization experience group, seems to highlight the resonating power of negative personal experiences with law enforcement. Law enforcement harassment was significantly related to perceptions in such a way that as law enforcement harassment incidents increase, the confidence that individuals have in law enforcement decreases in this study. In this study, on average, most of the individuals did not experience personal law enforcement harassment themselves, but rather had a family member undergo the experience. Feagin and Sikes (1994) espoused that a Black victim of law enforcement harassment is likely to share the experience with family members and friends, often to experience catharsis. One of the end results is a domino effect of pain and anguish that affects the psyche of others within the cultural group. This finding seems to be consistent with a number of research studies that have found that vicarious experience of law enforcement harassment has a negative influence on perceptions of law enforcement (Hurst and Frank, 2000; Rosenbaum et al., 2005). Finally, these findings may lend credence to the “negativity bias.” When people are faced with a mix of negative and positive events, the negative ones predominate in shaping one's thoughts and behavior. Baumeister et al. (2001) concluded that people more readily remember negative events than positive events.

These findings seem to highlight research that indicates that the type of contact with police is a major determinant of attitudes toward police (Correia et al., 1996; Weitzer and Tuch, 2002). Harassment and mistreatment can be interpreted differently by citizens and police, and what some citizens view as police harassment or brutality may be viewed by law enforcement as aggressive policing that is necessary for survival (Cox, 1996). Most citizens call police brutality an incident in which they have not been treated with the full rights and dignity of a citizen in a democratic society (Reiss, 1968). Any practice that degrades status, restricts freedom, annoys or harasses, or uses physical force is frequently seen as unnecessary and unwarranted by citizens (Reiss, 1968). One example of emotional and psychological abuse from officers could involve the usage of stereotypes and racial slurs. Labels for other groups such as drug dealers, homosexuals, prostitutes, and protestors may be used as well and prove to be derogatory in nature (Cox, 1996). Some of the reasons these differences may be occurring include African American citizens perceiving a lack of procedural justice, a high number of police contacts, perceived inequalities in racial fairness, race of the citizen, interactions initiated by the police, and lack of satisfaction with police related to crime in neighborhoods (Cheurprakobit, 2000; Tyler, 2001, 2005; Avdija, 2010; Callanan and Rosenberger, 2011). Finally, some studies argue that citizens make a distinction between general attitudes toward the police and the acknowledgment of police in personal neighborhoods (Schuck and Rosenbaum, 2005). These findings and discussions highlight the need to utilize specific as well as general items when assessing police victimization and perception as well as the exigency to consider how mediating factors are connected to police perceptions of performance.

Significant differences in perceptions of how police treat minorities across police victimization groups was not found in this study. The current authors and Chow (2010) found that the treatment of minorities scale consisted of two items. This finding should be cautiously interpreted given that it does consist of items and may not be as robust as other scales (Tabachnick and Fidell, 2007). This finding contradicts several studies that highlight that Blacks are more likely to feel they have not received procedural justice and are thus more likely to have a lower opinion of law enforcement (Tyler and Huo, 2002; Chow, 2008; Miller and Davis, 2008; Avdija, 2010). Given that the overall mean for the police treatment of minorities was high (*M* = 4.06) and most of the subgroups were within 0.50 scaled points, a lack of significant differences among groups may highlight that vicarious experiences are just as impactful as personal experiences of victimization. An individual's knowledge of another person's encounters with police may be internalized and “vicariously” experienced. Research has shown that these experiences may be communicated with other friends, family members, and acquaintances and have an effect that impacts subcultural beliefs about law enforcement (Harris, 2002).

Support for the third hypotheses was partially found in that satisfaction with police in most recent contact, crime reality television shows, and police excessive force media consumption were related to perceptions of police performance. Satisfaction with police in most recent contact and crime reality television show consumption was positively related to perceptions of police performance. Crime reality television show consumption was significantly related to confidence in that as crime reality-based show consumption increased, confidence in law enforcement performance seemed to increase in this study. This finding is consistent with Callanan and Rosenberger's (2011) research, but inconsistent with Edward's (2007) research that highlighted that crime reality television shows and crime drama shows had no significant relationship with attitudes toward law enforcement. Callanan and Rosenberger's (2011) research highlights that media related variables, such as crime drama consumption and crime reality television show consumption, have a significant relationship with perceptions of law enforcement when individuals are bereft of personal experiences with law enforcement. Fictional crime drama consumption was not related to African American students' perceptions of police performance. These findings may be related to the acquired education of the participants in this study. Mbuba (2010) posits that college students majoring in Criminal Justice gain specific knowledge that puts them in a better position to evaluate how well the social system performs based on an empirical and academic analysis; contrarily, non-criminal justice majors base their perceptions and attitudes toward the justice system on information gained from the mass media. Most of the individuals that participated in this study were African American students from social science courses who successfully completed introductory courses in sociology and/or psychology. The relationship between satisfaction with most recent police contact and perceptions of law enforcement performance seems to be consistent with research that emphasizes that positive perceptions tend to be experienced by African Americans when their interactions with law enforcement are respectful and non-threatening (Birzer, 2008). Though our findings have indicated that satisfaction with most recent contact with police is related to perceptions, there is research that highlights that positive contacts with police do not consistently translate into favorable attitudes about law enforcement (Leiber et al., 1998). A satisfying experience with police may be viewed as the exception rather than the norm, and preexisting experiences with law enforcement may influence the interpretation of a positive encounter with law enforcement (Brandl et al., 1994). These findings warrant the need to interpret this study's findings with caution and consider how satisfaction with law enforcement may be influenced by multiple factors.

Police excessive force media consumption was negatively related to perceptions of performance. In other words, the more participants watched non-fictional law enforcement brutality incidents, the more likely they were to experience decreased confidence in law enforcement. These findings seem to align with research that shows that citizens' perceptions of police are influenced by micro- and macro-level predictors (Weitzer and Tuch, 2005). This finding also seems to corroborate existing research that highlights African Americans as having less confidence in law enforcement following well-publicized law enforcement brutality events (Weitzer, 2002). Furthermore, the finding seems to support existing research that highlights that highly publicized law enforcement brutality incidents, such as Rodney King in 1991, can significantly alter the perceptions of law enforcement and how they treat minority groups (Jefferis et al., 1997). Continuous exposure to news reports on law enforcement abuse of authority (e.g., excessive physical force, corruption, and verbal abuse) is strongly related to perceptions of law enforcement racial bias by African Americans, Hispanics, and Whites. Furthermore, repeated exposure to media accounts of law enforcement abuse predicts that all racial groups will perceive law enforcement as operating in a discriminatory fashion and racially profile minorities (Weitzer and Tuch, 2005). These findings may be best explained by the vicarious experiences that individuals share within a cultural group. Though the viewer of these images is not suffering the mistreatment firsthand, the relatedness that the viewer has with the recipient of the excessive physical force may cause vicarious traumatization, which in turn affects cognitions about law enforcement. Due to the reality of these issues and how emotionally charged these incidents are, these flashbulb memories not only permeate the psyche of one individual, but they go on to have a multiplied effect within the cultural network of the viewer as the incident is processed and shared.

Finally, support for the final hypothesis was not found in that satisfaction with police in most recent contact, crime reality television, fictional crime drama consumption, and police excessive force media consumption were not related to perceptions of how police treat minorities. As stated earlier, these findings should be interpreted with caution given the two-item structure of the dependent variable. Though none of the predictors reached significance, police excessive force media consumption approached significance by 0.005 points, and perhaps this highlights the need to take other factors into consideration when assessing African American students' perceptions of the treatment of minorities. These other factors may include neighborhood of origin, socio-economic status, fear of police, and questionable police legitimacy (Hurst and Frank, 2000; Howell et al., 2004; Goldsmith, 2005; Avdija, 2010). Overall, the significant differences and relationships found in the analyses that included police performance and the lack of significance in the treatment of minorities models may highlight distinctions made in evaluating police. Comparing the mean descriptive statistic of police performance and police treatment of minorities highlights a moderate distinction that warrants exploration in future studies. Evaluations can be quite distinct based on general evaluations and specific evaluations (Schuck and Rosenbaum, 2005). This study seems to indicate that distinctions are made not in regard to locale only, but in regard to population or demographic being served by police.

### Limitations

One of the major limitations of this study was its relatively small sample size of African American males and sampling from a single institution. Furthermore, there was an oversampling of females who were generally younger, evidenced by the most common age of participant in this study being 19 years of age. Generalizability cannot be assumed to transfer to non-HBCUs given the specific focus of this study. Future directions should involve incorporating larger samples with equal portions of male and female students to provide stronger basis for predictive models. If possible, future studies should also include a non-HBCU control group to assess for significant differences that may exist between African American college students. Considerable thoughts should also be given to incorporating more background information in the analysis as it pertains to academic major and neighborhood of origin to assess how those factors are related to perceptions of police. Global items as well as specific items should be incorporated in an analysis of one's perception of police given distinctions that ethnic and racial groups may make when evaluating the police.

### Implications

Though this research focuses on the perceptions of police held by African American college students' and highlights some of the predictors, it should be noted that in terms of police and community relations the participants of this study are only one part of the dynamic. Any lasting change that will impact perceptions will involve all groups involved and will be a result of genuine and procedural changes in human relations. Police, minority groups, and the dominant group all have somewhat negative images of each other and each of these groups brings certain expectations to encounters. It is reasonable to expect that all participants in police interactions are subject to misinterpretations and misperceptions of one another's actions and intentions. Accurate depictions need to replace negative stereotypes in all groups, and there is a need for concerted effort toward mutual concern, understanding, and respect. Given that satisfaction with police was linked to increased confidence in police performance in this study, several suggestions could be employed by police. To improve police and community relations, campus police can do the following: establish reasonably well-publicized grievance procedures, hold public meetings to resolve issues, open the police department to public inspection, provide opportunities for citizens to ride with patrol officers, invite the public to participate in policy making, take prompt disciplinary procedures against officers who violate the law, and citizen police academies where citizens can learn more about police procedures (Cox and Fitzgerald, 1996).

These solutions are not meant to be exhaustive or perfect, and admittedly they are focused on one party since law enforcement cannot require minority citizens to engage in any intervention to improve police minority relations (Cox and Fitzgerald, 1996). Nevertheless, the police must take initiative in improving police and minority relations, even when it is counter to police subculture and encounters are unpleasant. It will be difficult to reconcile differences among groups if neither party understands the need for mutual respect, understanding, and cooperation. It is the authors' hope that the information presented could serve as fodder for further discussion on how to remedy the chronic strained interactions that continue to exist between law enforcement and African Americans with and without histories of police victimization.

## Ethics Statement

All researchers and authors associated with this study acted in accordance with institutional review board standards following approval of the project. Informed consent was sought and obtained from all research participants and confidentiality and anonymity was ensured for all participants in the study. All participants voluntarily participated in the study and were informed of their right to withdraw during any point of the study. The researchers and authors strove to avoid harm to all participants and report conclusions related to the variables in an independent and impartial manner.

## Author Contributions

All authors listed have made a substantial, direct and intellectual contribution to the work, and approved it for publication.

### Conflict of Interest Statement

The authors declare that the research was conducted in the absence of any commercial or financial relationships that could be construed as a potential conflict of interest.
